# Supplementary figures and images for: Robotic RNA extraction for SARS-CoV-2 surveillance using saliva samples
Source: PLoS One. 2021 Aug 5;16(8):e0255690. doi: 10.1371/journal.pone.0255690 (PMC8341588; doi:10.1371/journal.pone.0255690)

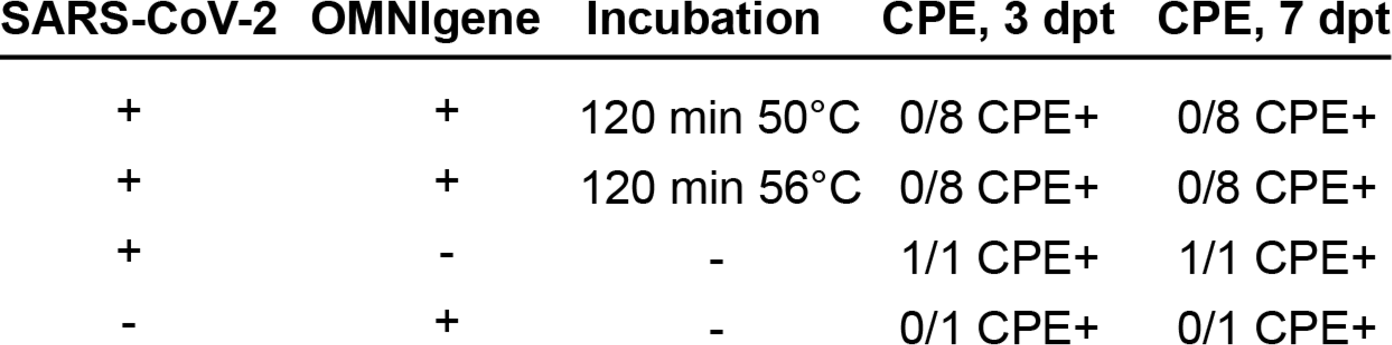

Supplement: S1 Fig — Cultured SARS-CoV-2 (3.16x10 TCID50/ml) was mixed 1:1 with OMNIgene solution present in OM-505 collection tubes to test incubation conditions that inactivate viral replication. Samples were either incubated at 50°C or 56°C for the indicated length of time before being applied to Vero-E6 cells. Cytopathic effect (CPE) was quantified at 3 and 7 days post treatment (dpt). (TIF) [file pone.0255690.s001.tif]

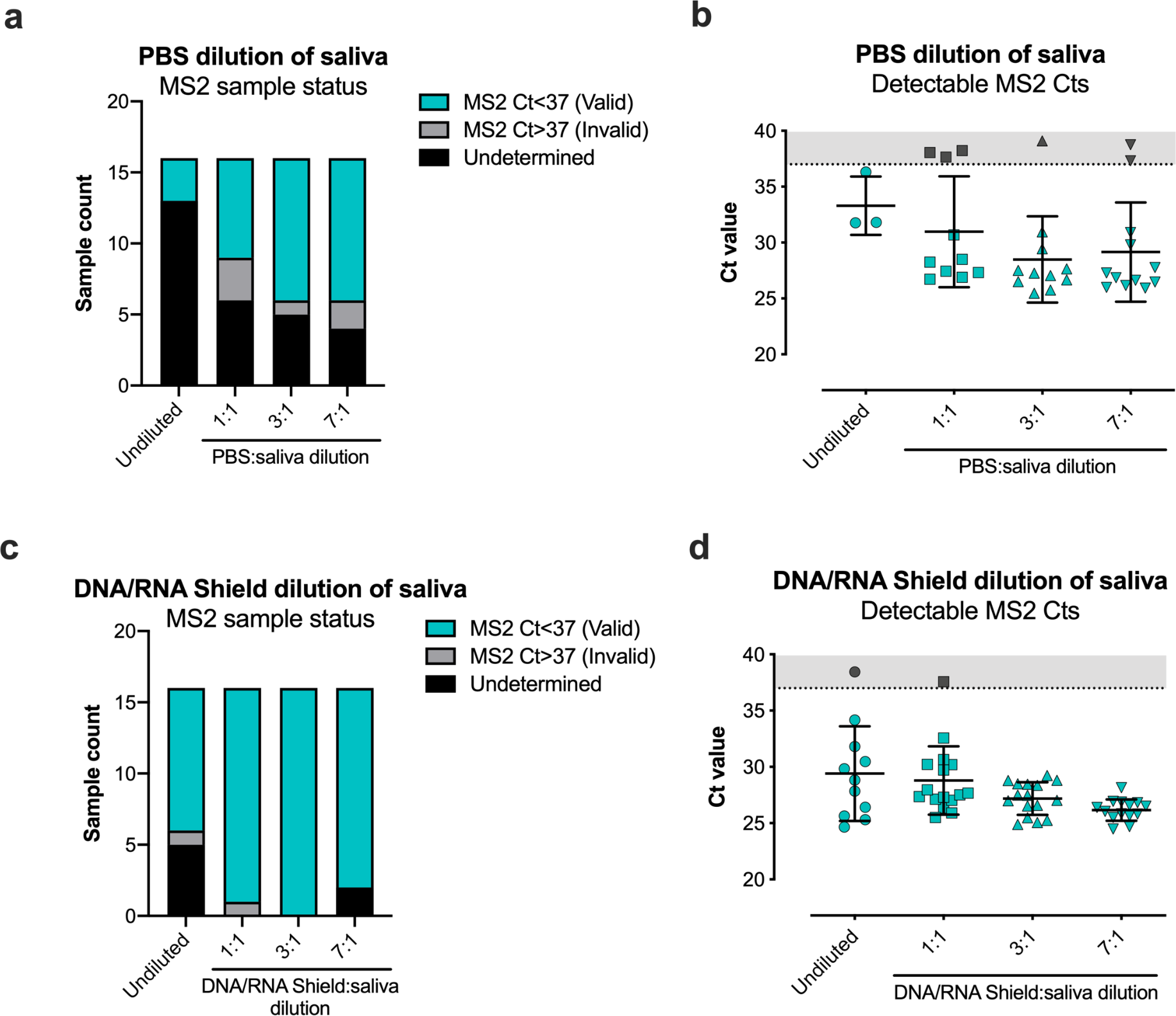

Supplement: S2 Fig — Saliva samples previously reported “specimen insufficient” in the IGI FAST study were diluted in either phosphate buffered saline (PBS) or 2x DNA/RNA Shield and the RT-qPCR detection of spiked-in MS2 was used to quantify sample extraction efficiency. a, Sample status after qRT-pCR for MS2 after serial dilution in PBS. The same set of 16 saliva samples were used for each dilution condition. b, MS2 Ct values for samples diluted in PBS. c, Sample status after qRT-pCR for MS2 after serial dilution in DNA/RNA Shield. The same set of 16 saliva samples were used for each dilution condition (distinct from the samples used for a). d, MS2 Ct values for samples diluted in DNA/RNA Shield. For b and d, the mean and standard deviation are plotted for each group and invalid MS2 Ct>37 are indicated in gray. (TIF) [file pone.0255690.s002.tif]

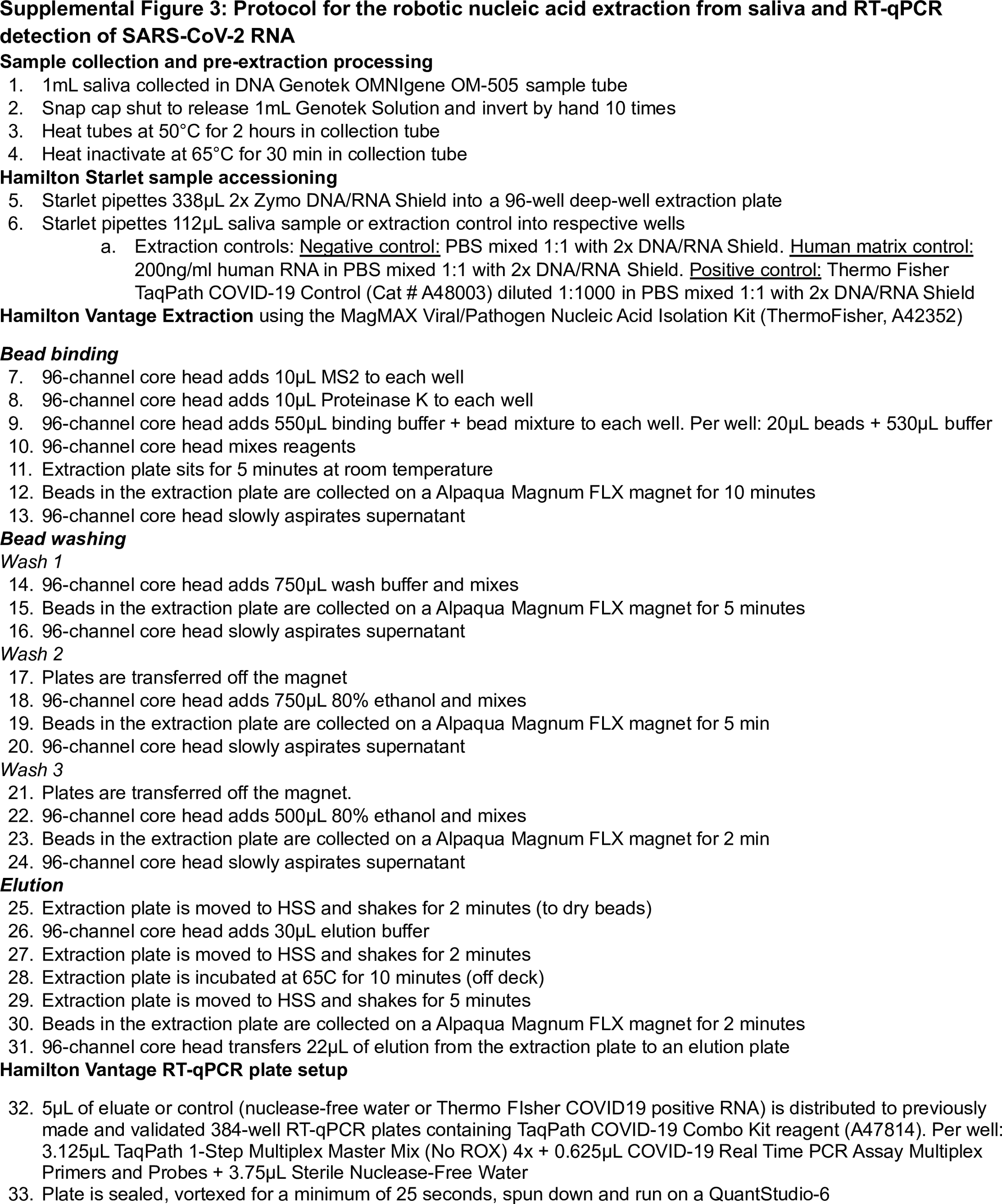

Supplement: S3 Fig — (TIF) [file pone.0255690.s003.tif]

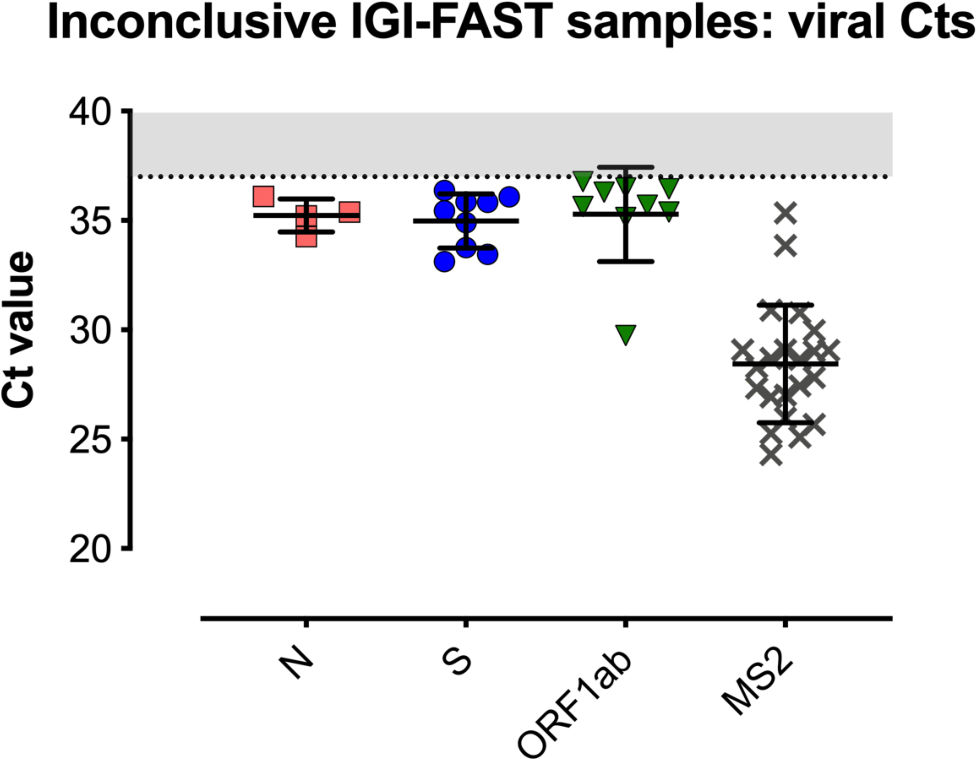

Supplement: S4 Fig — Viral and MS2 Cts for IGI FAST saliva samples with an inconclusive result (one viral gene and MS2 detected at a Ct value <37). Mean and standard deviation are plotted for each group. Gray shading indicates Ct values >37. (TIF) [file pone.0255690.s004.tif]

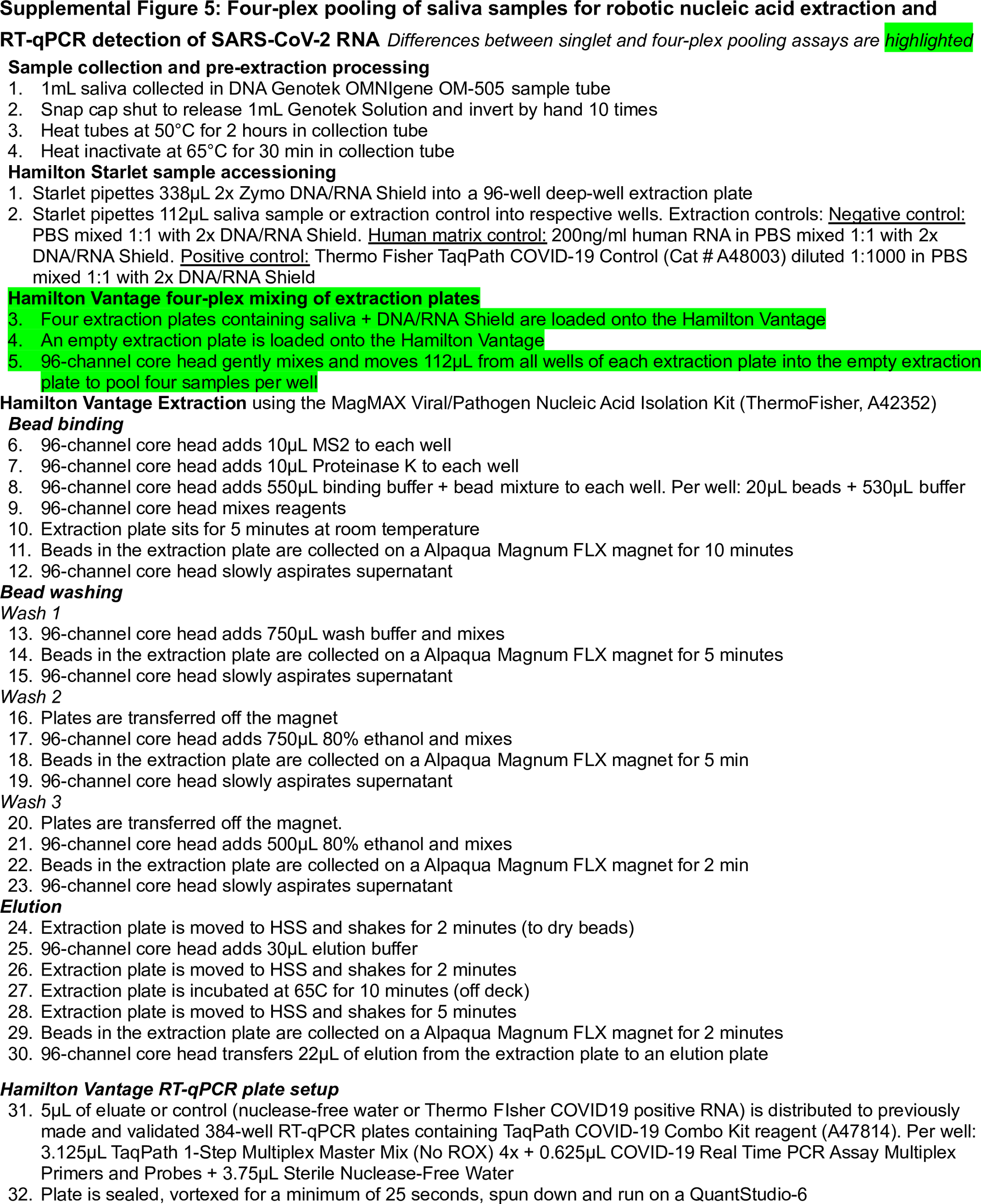

Supplement: S5 Fig — (TIF) [file pone.0255690.s005.tif]

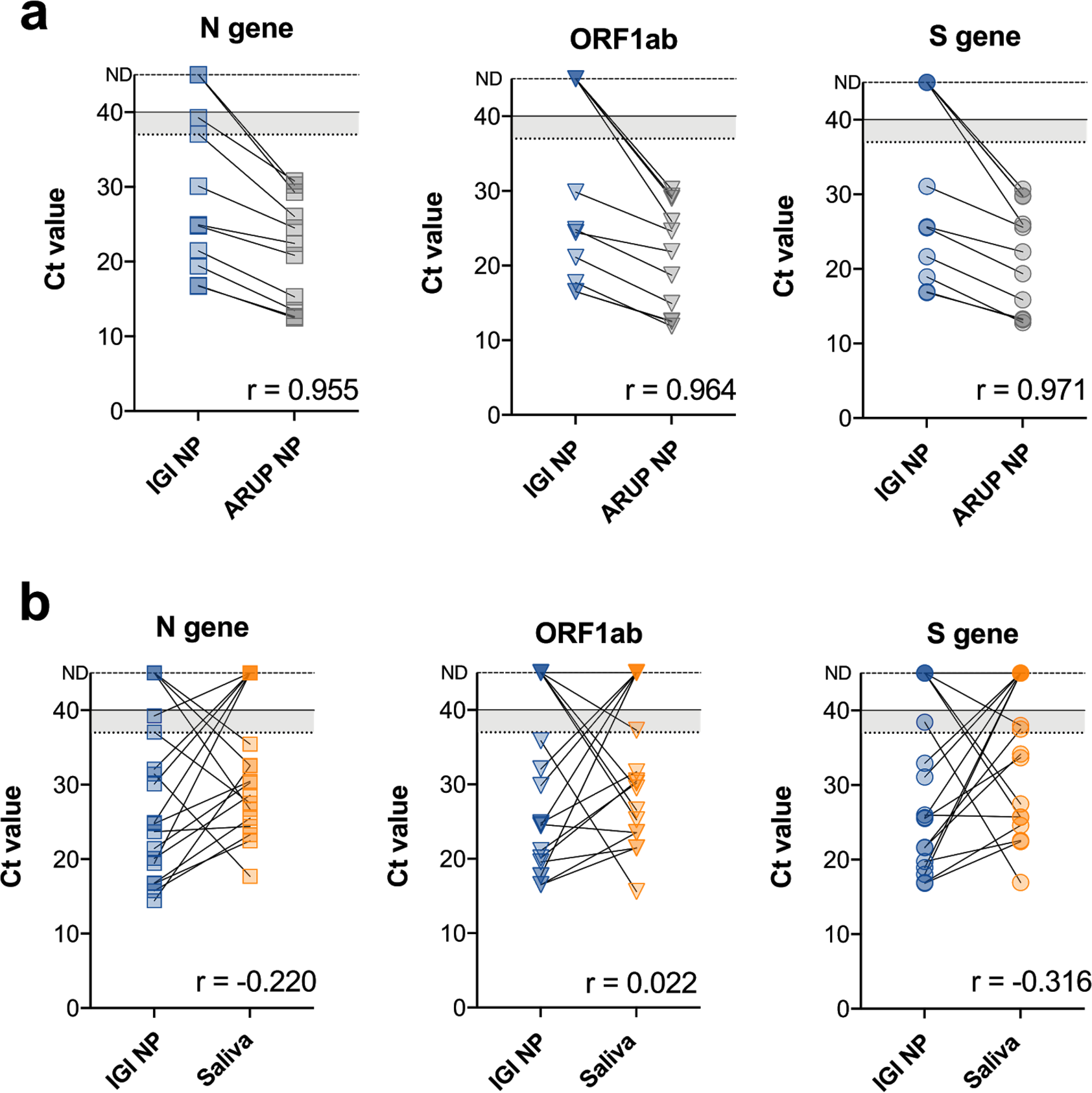

Supplement: S6 Fig — a, Ct values for NP specimens tested by ARUP and IGI using the same primer-probe pairs are plotted with paired results connected by a line. b, Ct values for paired NP and Saliva specimens processed by IGI are connected by a line showing only those with paired results in the two assays. Ct values above the 37 cutoff (dotted line) are shaded in gray. PCR was run for 40 cycles. Undetected Ct values are plotted at the top of each graph and designated by “ND”, not detected. Pearson correlation (r) was calculated for each gene between the two indicated assays where undetected Ct values were left blank (no value assigned). (TIF) [file pone.0255690.s006.tif]
